# Benefits and risks of antiplatelet therapy for moyamoya disease: a systematic review and meta-analysis

**DOI:** 10.3389/fneur.2023.1132339

**Published:** 2023-06-20

**Authors:** Tingting Liu, Mingzhen Qin, Xuejiao Xiong, Tingting Li, Luda Feng, Xinxing Lai, Ying Gao

**Affiliations:** ^1^Institute for Brain Disorders, Beijing University of Chinese Medicine, Beijing, China; ^2^Department of Neurology, Dongzhimen Hospital, Beijing University of Chinese Medicine, Beijing, China; ^3^Beijing University of Chinese Medicine, Beijing, China; ^4^Dongfang Hospital, Beijing University of Chinese Medicine, Beijing, China; ^5^Chinese Medicine Key Research Room of Brain Disorders Syndrome and Treatment of the National Administration of Traditional Chinese Medicine, Beijing, China

**Keywords:** moyamoya disease, antiplatelet, benefits, risks, systematic review, meta-analysis

## Abstract

**Background:**

Moyamoya disease (MMD) is a leading cause of stroke in children and young adults, whereas no specific drugs are available. Antiplatelet therapy (APT) has been considered a promising treatment option, but its effectiveness remains controversial. Therefore, we aimed to comprehensively evaluate the benefits and risks of APT for MMD.

**Methods:**

We systematically searched PubMed, Embase, and Cochrane Library electronic databases from their inception to 30 June 2022 and conducted a systematic review. All-cause mortality was taken as the primary outcome.

**Results:**

Nine studies that enrolled 16,186 patients with MMD were included. The results from a single study showed that APT was associated with lower mortality [hazard ratio (HR) = 0.60; 95% confidence interval (CI) (0.50–0.71); *p* < 0.01] and improved bypass patency after surgical revascularization [HR = 1.57; 95% CI (1.106–2.235); *p* < 0.05]. The results of the meta-analysis showed that APT reduced the risk of hemorrhagic stroke [HR = 0.47; 95% CI (0.24–0.94); *p* < 0.05] but neither reduced the risk of ischemic stroke [HR = 0.80; 95% CI (0.33–1.94); *p* = 0.63] nor increased the proportion of independent patients [RR = 1.02; 95% CI (0.97–1.06); *p* = 0.47].

**Conclusion:**

Current evidence showed that APT was associated with a reduced risk of hemorrhagic stroke in MMD patients but did not reduce the risk of ischemic stroke or increase the proportion of independent patients. There was insufficient evidence about the benefit of APT on survival and postoperative bypass patency after surgical revascularization. However, the results should be interpreted cautiously because of the limited number of studies.

**Systematic review registration:**

https://www.crd.york.ac.uk/prospero/.

## 1. Introduction

Moyamoya disease (MMD), also known as abnormal vascular network disease at the base of the skull, is characterized by progressive stenosis or occlusion of the ends of the bilateral internal carotid arteries as well as the proximal anterior and middle cerebral arteries, accompanied by the development of small collateral vascular networks (1–3). MMD is the leading cause of stroke in children and young adults in East Asian countries, leading not only to irreversible neurological deficits and death but also to severe disease and economic burden (3–5).

MMD treatment aims to prevent the progression of the primary disease process and reduce the risk of ischemic or hemorrhagic stroke (6). Despite the cumulative understanding of the genetic and pathophysiological basis of MMD, including mutations in RNF213 and increased activity of various growth factors, there are no specific and effective therapeutic drugs (1, 3, 7, 8). Growing evidence suggests that intraluminal thrombosis is an important pathological feature of MMD; therefore, antiplatelet therapy (APT) is considered a promising treatment option for MMD (3, 9, 10). Studies have shown that APT can reduce the risk of ischemic stroke in patients with MMD and maintain a smooth flow of remodeled blood vessels (11, 12). It can be used in the acute phase of ischemic stroke and the chronic phase of stroke prevention and also in the perioperative phase of surgical treatment for MMD (13). However, some experts are concerned that APT may not reduce the incidence of ischemic stroke and may increase the risk of intracranial hemorrhage (11). Furthermore, the effectiveness of APT in patients with MMD has been inconsistent or contradictory in several studies.

Thus, to address this therapeutic dilemma, this systematic review and meta-analysis aimed to comprehensively evaluate the benefits and risks of APT for MMD and provide evidence to guide decision-making in the use of antiplatelet drugs for MMD.

## 2. Methods

The protocol of this study has been registered in PROSPERO (registration number: CRD42022319700). This systematic review and meta-analysis were reported in accordance with the Preferred Reporting Items for Systematic Reviews and Meta-analyses (PRISMA) guidelines (14).

### 2.1. Search strategy and study screening

We comprehensively searched the PubMed, Embase, and Cochrane Library electronic databases without language limitations from their inception to 30 June 2022. All searches were conducted by combining the free text and MeSH terms, containing “moyamoya disease,” “progressive intracranial occlusive arteropathy,” “platelet aggregation inhibitors,” “antiplatelet,” “aspirin,” “clopidogrel,” “ticagrelor,” and “cilostazol” (details are presented in Supplementary Table 1). In addition, we hand-searched the World Health Organization International Clinical Trials Registry Platform and 18 first-level clinical trial registration platforms, including ClinicalTrials.gov, Chinese Clinical Trial Registry, Clinical Research Information Service-Republic of Korea, and Japan Registry of Clinical Trials. Two reviewers (M.Q. and X.X.) independently screened titles and abstracts and selected potential full texts for further analysis. Studies fulfilling our predefined eligibility criteria were included in the review. Any disagreements were resolved through discussion or consultation with a third reviewer (T.L.).

### 2.2. Inclusion and exclusion criteria

The detailed inclusion criteria were as follows: (a) definitive diagnosis of MMD; (b) intervention or exposure to APT, regardless of whether patients underwent surgical revascularization; (c) outcomes during the follow-up period included at least one of the following: all-cause mortality, ischemic stroke, hemorrhagic stroke, postoperative bypass patency, and proportion of independent patients (proportion of independent patients was defined as a modified Rankin scale score of 0, 1, or 2); and (d) randomized controlled trials (RCT) or cohort studies. To address heterogeneity secondary to differences in follow-up duration, we reported time-to-event outcomes as hazard ratios (HRs) with 95% confidence intervals (CIs). We reported dichotomous outcomes (i.e., the proportion of independent patients) as risk ratios (RRs) with 95% CIs. Non-human studies, reviews, and commentary papers were excluded.

### 2.3. Data extraction

Data were independently extracted by two reviewers (M.Q. and X.X.) using a preformulated data collection form that included (a) the article's author and publication year; (b) study characteristics, including study site, sample size, main population characteristics, follow-up period, APT regimens, and outcomes; and (c) adjusted variables. If multiple analysis models were presented in the study, the outcomes were extracted from the most fully adjusted model. For each study, all relevant data were extracted from the tables, figures, text, and supplementary materials. If data on HR and 95% CI were not directly available, we performed transformations or calculations with other relevant data according to the practical guidelines suggested by Tierney et al. (15). When necessary, we used the software OriginPro (https://www.originlab.com/) to obtain relevant data from Kaplan–Meier survival plots.

### 2.4. Quality assessment

The quality of randomized controlled trials was assessed using the Cochrane risk of bias assessment tool, which consists of six components, including selection bias, performance bias, detection bias, follow-up bias, reporting bias, and other biases (16). The quality of cohort studies was assessed using the Newcastle–Ottawa Scale, which has three components including selection, comparability, and outcomes. According to the scale score (ranging from 0 to 9 points), the quality of cohort studies was divided into high (7–9 points), fair (4–6 points), or low (< 4 points) (17).

### 2.5. Statistical analysis

Statistical analysis was performed using RevMan 5.4 software, where the data used were adjusted as much as possible. In each study, HR and 95% CI were converted by using their natural logarithms, and then SEs were calculated from these logarithmic numbers. Finally, log HRs and SEs were combined using the inverse variance approach. Relative risk (RR) was calculated from the number of events and participants. Heterogeneity between studies was calculated using *I*^2^ statistics. When *I*^2^ was < 25%, the pooled effect size was calculated using the fixed-effect model, and when *I*^2^ was 25% or greater, the random-effect or qualitative analysis model was used. All tests were two-sided, and statistical significance was defined as a two-tailed *p-*value of < 0.05.

Subgroup and sensitivity analyses were performed to identify the source of heterogeneity, while *I*^2^ was ≥ 25%. The subgroup analyses were performed according to race (non-Asian and Asian patients) on whether patients underwent surgical revascularization, type and daily dose of antiplatelet drugs, duration of drug use, and study quality. The sensitivity analysis was performed by sequentially excluding each study to calculate the pooled effect size of the remaining studies. Additionally, a sensitivity analysis was conducted to test the stability of the results using the random-effects model, while 0 < *I*^2^ < 25%. Publication bias was assessed using funnel plots and Egger's regression asymmetry test (*p* < 0.05 was considered representative of statistically significant publication bias). A descriptive analysis was performed if there were too few studies to conduct a meta-analysis or if the clinical heterogeneity was too large.

## 3. Results

### 3.1. Search results

**Figure 1:**
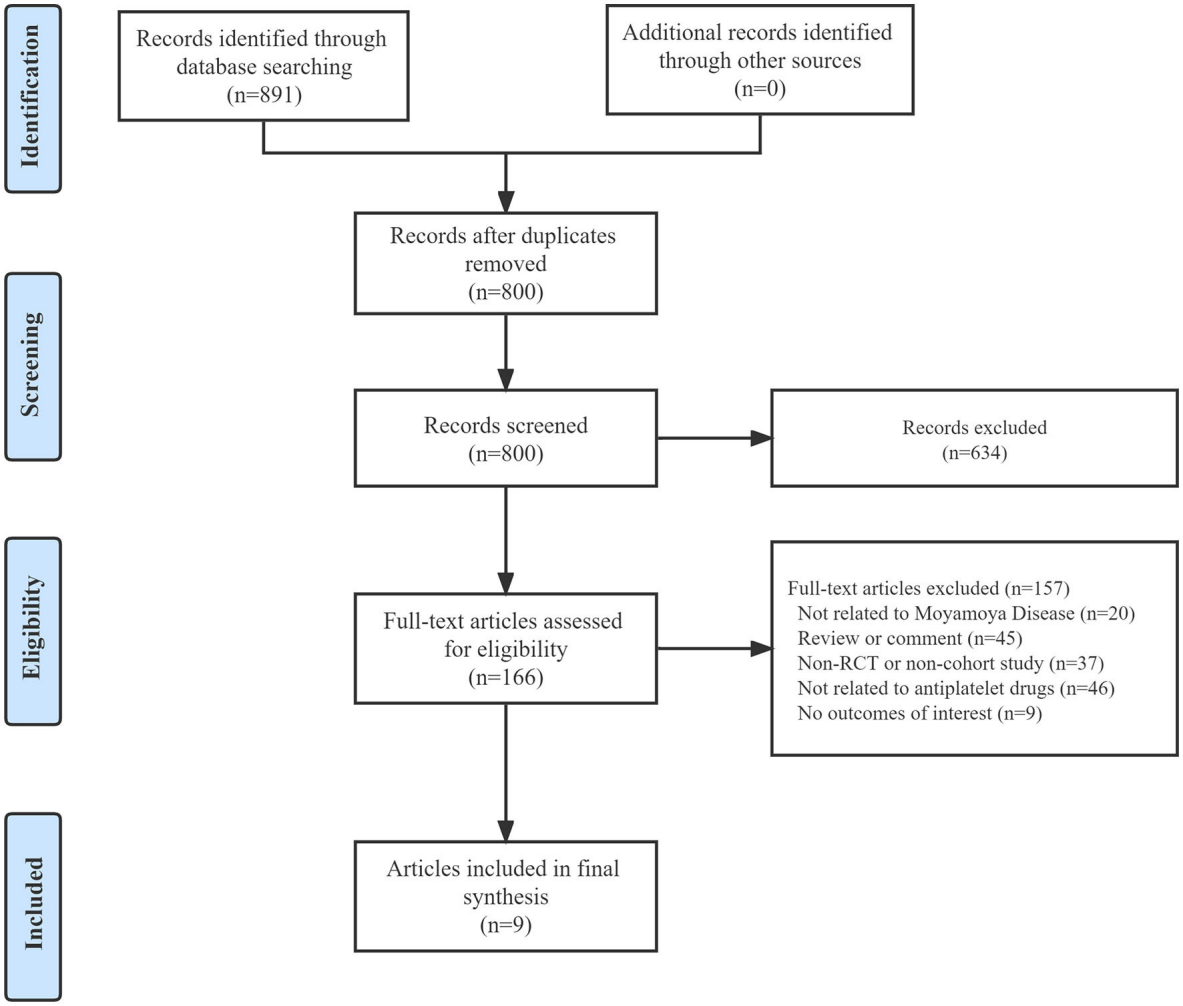
Flow diagram of study selection.

A total of 891 articles were obtained through our systematic search of three electronic databases, of which 91 were excluded owing to duplication. After screening titles and abstracts, 166 potential articles with full text were retrieved. Among them, 20 articles were not related to MMD, 45 were reviews or commentaries, 37 were non-RCT or non-cohort studies, 46 were not related to APT, and 9 had no outcome of interest. Eventually, nine articles were included in the final dataset after a full-text review, and all the articles were cohort studies (12, 18–25). The literature search and article selection are outlined in Figure 1. In addition, by manually searching the clinical trial registration platforms, we found no studies that met our inclusion criteria.

### 3.2. Characteristics of the included studies

Overall, nine studies involving 16,186 participants were included [10,535 (65.09%) women]. The characteristics of the included studies are listed in Table 1. All studies were conducted in Asia, including China, South Korea, and Japan, which could be related to the incidence of the Moyamoya and are the so-called east-high and west-low global distribution (3). The primary method of data collection for the original studies was to extract information from electronic medical records, and the average follow-up ranged from 6 to 218 months. Three studies included patients who underwent surgical revascularization (19, 24, 25). Five studies performed propensity score matching, one of which performed propensity score matching only for the outcome of analyzing bypass patency rates (12, 18, 19, 23, 25). Five studies reported details of the type of antiplatelet drugs, and all of them had at least one oral antiplatelet drug (12, 18–20, 25). Of note, three studies specifically reported medication course: 19,251.5 patient-years, 72.5 ± 48.4 months, and 2.7 ± 1.3 years, respectively (18–20).

**Table 1:** Characteristics of included studies.

**References**	**Data source, country**	**Main population characteristics**	**Number. of patients (female)**	**Age, years (mean ±SD)**	**Follow-up, (mean ±SD)**	**Mean duration of APT**	**Type of APT**	**Outcome**	**Outcome assessment**	**Adjusted variables**
Xiang et al. (25)	One Chinese hospitals, China	Undergone surgical revascularization	(PSM) APT: 95 (54) No APT: 95 (57)	45.01 ± 9.3 45.38 ± 9.57	(Entire) 2.7 y	NA	Aspirin	mRS	Doctors who were unaware of the study-group assignments	Sex, age, risk factors, individual comorbidities, symptoms, hospitalization days, mRS at admission, Suzuki stage, and rate of incision healing.
Seo et al. (18)	NHIS database, Korean	Confirmed first-ever diagnosis of MMD	(PSM) APT: 7,144 (4,624) No APT: 7,144 (4,719)	(Entire) 37.6 ± 19.9	(Entire) 6.3 y	(Entire) 19,251.5 patient-years	Aspirin, clopidogrel, cilostazol, others (composed of triflusal and ticlopidine), or two	Mortality	Via death dates recorded in NHIS	Age, sex, cancer, hypertension, diabetes, atrial fibrillation, cerebral revascularization surgery, and dyslipidemia.
Pang et al. (20)	Medical database of Seoul National University Hospital, Korean	Initial clinical follow-up without surgery of at least 6 months or more	APT: 121 (88) No APT: 122 (94)	44.2 ± 12.0 43.3 ± 10.7	72.5 ± 48.4 m 51.5 ± 35.0 m	72.5 ± 48.4 m	Potency 1: pentoxifylline, ibudilast, and triflusal Potency 2: acetylsalicylic acid, cilostazol, dipyridamole, and sarpogrelate Potency 3: ticagrelor and clopidogrel Potency 4: dual and including at least one of Potency 3	IS; ICH	NA	NA
Lu et al. (19)	Two Chinese hospitals, China	Undergone surgical revascularization	(PSM) APT: 78 (39) No APT: 78 (42)	37.5 ± 9.6 37.6 ± 8.9	(Entire) 2.7 ± 1.3y	(Entire) 2.7 ± 1.3y	Aspirin	Bypass patency	Two independent radiology-trained neurosurgeons	Age, sex, Suzuki stage, posterior circulation involvement, smoking, and diabetes.
Ye et al. (12)	Eight Chinese teaching hospitals, China	Clinical presentations of TIA or IS	(PSM) APT: 53 (22) Conservative: 53 (24)	48 ± 11 48 ± 13	35.6 ± 20.0 m 32.2 ± 14.1 m	NA	Aspirin; clopidogrel; aspirin and clopidogrel for the first 3 weeks, followed by aspirin.	IS	Data coordinators who were unaware of the study-group assignments	Age, sex, alcohol consumption, hypertension, diabetes, smoking, dyslipidemia, mRS, a history of stroke or TIA, a family history of MMD, TIA, IS, lacunar infarction, steno-occlusive change, Suzuki stage, and intracranial aneurysm.
Nam et al. (21)	Two hospitals, Korean	Without surgical revascularization	(Entire) 84 (58)	(Entire) 44	(Entire) 79.9 (65.8–103.8) m	NA	NA	IS	Neuroradiologists who were blinded to findings	Initial presentation (IS), Ivy sign
Zhao et al. (24)	Beijing Tiantan Hospital, China	Undergone surgical revascularization	APT: 59 (28) No APT: 138 (73)	38.7 37.3	NA	NA	NA	mRS	NA	NA
Yamada et al. (22)	30 core facilities, Japan	NA	APT: 191 (121) No APT: 153 (110)	34.9 ± 18.2 30.3 ± 18.5	(Entire) 10 y	NA	NA	IS; ICH	NA	NA
Onozuka et al. (23)	Nationwide registry data, China	Non-hemorrhagic MMD	(PSM) APT: 289 (196) No APT: 289 (186)	30.9 ± 18.8 31.3 ± 18.9	NA	NA	NA	mRS	Neurologists who were blinded to treatment	Age, sex, BMI, smoking, hypertension, diabetes, hyperlipidemia, and emergency

APT, antiplatelet therapy; BMI, body mass index; IS, ischemic stroke; ICH, intracerebral hemorrhage; m, months; MMD, moyamoya disease; mRs, modified Rankin scale; NA, not available; NHIS, national health insurance service database; PSM, propensity score matching; TIA, transient ischemic attack; SD, standard deviation; STA-MCA, superficial temporal artery and middle cerebral artery; y, years.

### 3.3. Quality of studies

Four studies were rated nine points according to the Newcastle–Ottawa scale, except for the studies by Xiang et al., Pang et al., Zhao et al., Yamada et al., and Onozuka et al. The study by Onozuka et al. was rated eight points for insufficient follow-up; the study by Xiang et al. was rated eight points because of poor representation of the exposed group; the study by Pang et al. was rated seven points owing to poor representation of the exposed group and insufficient outcome assessment; the study by Zhao et al. was rated six points owing to poor representation of the exposed group, insufficient outcome assessment, and insufficient follow-up; and the study by Yamada et al. was rated five points owing to poor comparability between groups, insufficient outcome assessment, and insufficient follow-up (20, 22–25). All studies were of high quality, except for two with a fair quality rating. A detailed evaluation of the bias risk of all studies is shown in Table 2.

**Table 2:** Evaluation of methodological quality of included studies.

**References**	**Selection**				**Comparability**	**Outcome**			**Total stars**
	**Representativeness of cohort**	**Selection of the non-exposed cohort**	**Ascertainment of exposure**	**Outcome of interest was not present at start of study**	**Comparability of Cohorts on the basis of the design or analysis**	**Assessment of outcome**	**Was follow-up long enough for outcomes to occur**	**Adequacy of follow up of cohorts**	
Xiang et al. (25)		^*^	^*^	^*^	^**^	^*^	^*^	^*^	8
Seo et al. (18)	^*^	^*^	^*^	^*^	^**^	^*^	^*^	^*^	9
Pang et al. (20)		^*^	^*^	^*^	^**^		^*^	^*^	7
Lu et al. (19)	^*^	^*^	^*^	^*^	^**^	^*^	^*^	^*^	9
Ye et al. (12)	^*^	^*^	^*^	^*^	^**^	^*^	^*^	^*^	9
Nam et al. (21)	^*^	^*^	^*^	^*^	^**^	^*^	^*^	^*^	9
Zhao et al. (24)		^*^	^*^	^*^	^**^			^*^	6
Yamada et al. (22)	^*^	^*^	^*^	^*^			^*^		5
Onozuka et al. (23)	^*^	^*^	^*^	^*^	^**^	^*^		^*^	8

^*^represents score.

### 3.4. Primary outcomes

Only one study with a mean follow-up of 6.3 years assessed the effect of antiplatelet drugs on mortality in patients with MMD, including 9,154 patients taking antiplatelet medication at least once after MMD diagnosis and 16,284 never taking antiplatelet medication (18). A propensity score matching analysis showed that any antiplatelet use was associated with a reduced risk of death [HR = 0.60; 95% CI (0.50–0.71); *p* < 0.01]. The types of antiplatelet drugs in this study included aspirin, clopidogrel, cilostazol, triflusal, and ticlopidine. A further subgroup analysis showed that cilostazol, a phosphodiesterase inhibitor, was associated with greater reductions in mortality than the other antiplatelet drugs, which could be related to two potentially favorable properties of phosphodiesterase inhibitors in MMD treatment, including a lower risk of intracranial hemorrhage and improved collateral blood flow with the aid of the vasodilation effect.

### 3.5. Secondary outcomes

#### 3.5.1. Ischemic stroke

**Figure 2:**
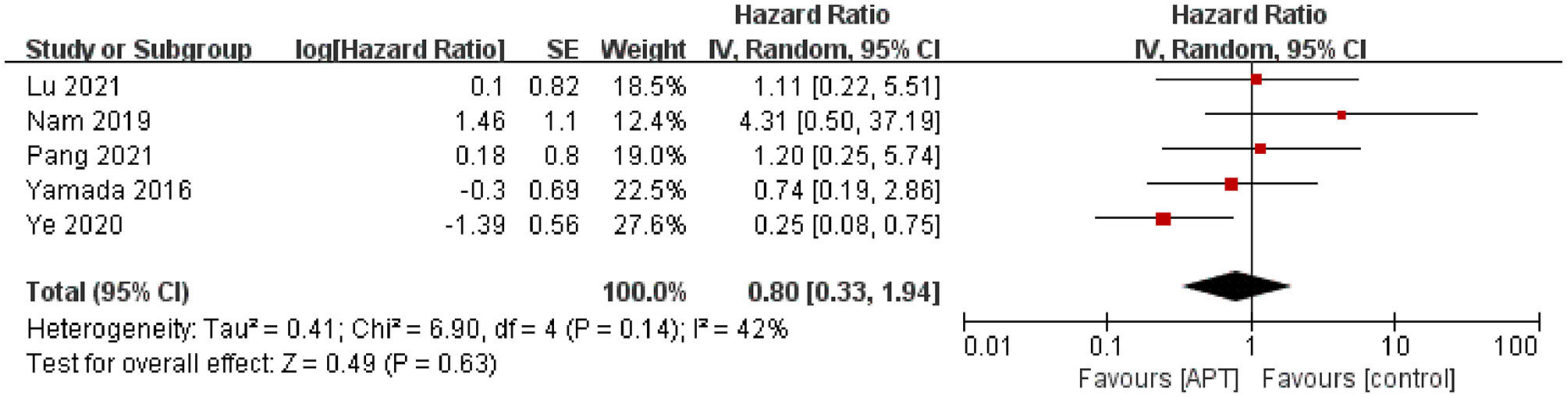
Forest plot of ischemic stroke.

Five studies assessed the risk of ischemic stroke during the follow-up period (12, 19–22). After testing for heterogeneity (*p* = 0.14; *I*^2^ = 42%), the pooled results using the random-effect model showed no evidence to support that APT reduces ischemic stroke in patients with MMD [HR = 0.80; 95% CI (0.33–1.94); *p* = 0.63; Figure 2]. Simultaneously, we performed subgroup analyses as planned. Because of limited data, we only performed subgroup analyses of whether patients underwent surgical revascularization and study quality, but we did not identify sources of heterogeneity (Supplementary Figures 1, 2). In terms of the subgroup analysis of revascularization, five studies were divided into three subgroups: three in the non-revascularization group, one in the revascularization group, and one in the unknown group. The pooled results were HR 0.89, 95% CI 0.18–4.5 for the non-revascularization group, HR 1.11, 95% CI 0.22–5.51 for the revascularization group, and HR 0.74, 95% CI 0.19–2.86 for the unknown group. In addition, sensitivity analyses were performed by excluding the studies individually. After excluding the study by Ye et al. (12), heterogeneity was significantly reduced (*p* = 0.60; *I*^2^ = 0), and pooled results from the fixed-effect model showed that APT did not reduce the risk of ischemic stroke in patients with MMD [HR = 1.18; 95% CI (0.53–2.64); *p* = 0.68; Supplementary Figure 3]. The results were consistent with those before the sensitivity analysis, indicating the robustness of the results.

#### 3.5.2. Hemorrhagic stroke

**Figure 3:**
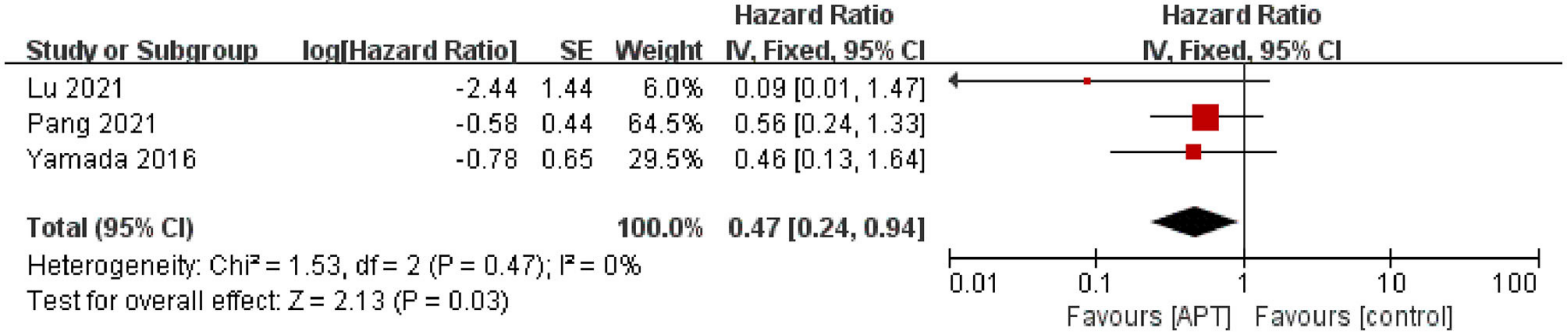
Forest plot of hemorrhagic stroke.

Three studies assessed the risk of ischemic stroke during the follow-up period (19, 20, 22). After testing for heterogeneity test (*p* = 0.47; *I*^2^ = 0), the pooled results using fixed-effect models showed that APT could reduce the risk of hemorrhagic stroke compared with the non-APT group [HR = 0.47; 95% CI (0.24–0.94); *p* < 0.05; Figure 3]. The random-effects model was used to assess the stability of the results, and the results were not significantly altered (Supplementary Figure 4).

#### 3.5.3. Bypass patency rate

One study used the bypass patency rate after surgical revascularization as an outcome measure during follow-up, determined using digital subtraction angiography/magnetic resonance angiography/computed tomography angiography by two independent radiology-trained neurosurgeons (19). All patients with ischemic MMD underwent superficial temporal artery-middle cerebral artery bypass. For patients without obvious intracranial hemorrhage on computed tomography scan 4 h after surgery, aspirin (100 mg once daily) was started the day after surgery, and patients were divided into two groups based on whether aspirin was continued after surgery. The APT group continued to take aspirin (100 mg once daily) after discharge until the last follow-up, while the non-APT group stopped taking aspirin within 1 month after discharge. The mean follow-up time was 2.7 ± 1.3 years. After propensity score matching, the results showed better bypass patency rates in the APT group [HR = 1.57; 95% CI (1.106–2.235); *p* < 0.05].

#### 3.5.4. Proportion of independent patients

**Figure 4:**
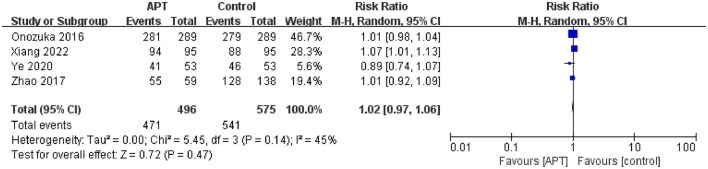
Forest plot of independent patients.

Four studies compared the efficacy of the APT and non-APT groups on the proportion of independent patients (12, 23–25). After testing for heterogeneity (*p* = 0.14; *I*^2^ = 45), pooled results using random-effects models indicated that APT was not associated with independent functional outcomes in patients with MMD [RR = 1.02; 95% CI (0.97–1.06); *p* = 0.47; Figure 4]. At the same time, we only performed subgroup analyses of whether patients underwent surgical revascularization and study quality; however, we did not identify sources of heterogeneity (Supplementary Figures 5, 6). Sensitivity analysis showed that after excluding the study by Xiang et al. (25), APT was not associated with independent functional outcomes in patients with MMD [RR = 0.99; 95% CI (0.96–1.03); *p* = 0.69; Supplementary Figure 7]. The results were consistent with those before the sensitivity analysis, indicating the robustness of the results.

### 3.6. Bias of publication

Owing to the small number of included studies (only nine), no further funnel plot analysis was performed to assess publication bias.

## 4. Discussion

### 4.1. Summary of the evidence

To the best of our knowledge, this was the first systematic review and meta-analysis to comprehensively evaluate the benefits and risks of APT in MMD. Nine studies with 16,186 patients with MMD were included. Moyamoya syndrome was not included in this systematic review. All included studies were from Asia. Regarding population characteristics of MMD, three studies included only patients who underwent revascularization (19, 24, 25), one study included only patients who did not undergo revascularization (21), and one study only included patients who did not undergo surgery for at least 6 months during the initial clinical follow-up (20). Five studies reported specific antiplatelet regimens, and all studies used at least one oral antiplatelet drug (12, 18–20, 25). The follow-up time of all studies was 6–218 months. The results of a single study showed that APT was associated with improved survival in patients with MMD and increased bypass patency after surgical revascularization. The results of the meta-analysis showed that APT could reduce the risk of hemorrhagic stroke but neither reduced the risk of ischemic stroke nor increased the proportion of independent patients. Because of limited data, we only performed subgroup analyses for two outcomes, namely, ischemic stroke and the proportion of independent patients. There was no evidence to support the effectiveness of APT for either outcome. The sensitivity analysis showed that the results were relatively robust.

Intraluminal thrombosis is an important pathologic feature of MMD. The intraluminal thrombus can block cerebral arteries *in situ* or cause arterial–arterial embolism, resulting in distal arterial occlusion (3, 9, 10). Antiplatelet drugs block platelet function via multiple pathways, showing antithrombotic effects and ultimately improving outcomes in patients with MMD. Our findings suggested that APT was associated with improved survival and bypass patency, which was supported by one study, respectively. High-quality large prospective cohort studies or randomized controlled trials are required for further validation. However, our results did not show that APT reduced the incidence of ischemic stroke and increased the proportion of independent patients. We speculated that this may be related to the fact that the included patients with MMD were mostly hemodynamically stable and therefore had a low rate of ischemia.

The safety of APT in patients with MMD has been a concern because APT may increase the risk of intracranial hemorrhage, especially in patients with choroidal collaterals. Previous studies showed that choroidal collaterals are a recently well-known risk factor for rebleeding and *de novo* bleeding in MMD patients (11, 26, 27). Interestingly, our findings showed that APT did not increase the risk of intracranial hemorrhage and even decreased the risk of subsequent hemorrhagic stroke, which to some extent, indicated the safety of APT for patients with MMD. Despite these promising findings, this result should be treated with caution as the results from one of the original studies were only from a univariate analysis of the ischemic subgroup (22).

According to the guidelines, surgical revascularization is recommended for patients with MMD in a non-emergency state, with obvious ischemic symptoms and no surgical contraindications, while non-surgical treatment is recommended for patients with mild ischemic symptoms and/or surgical contraindications. Considering the differences in the characteristics of the non-surgical population and the surgical population, we tried to conduct a subgroup analysis according to whether surgery was performed when assessing the benefits and risks of APT. The results showed that APT neither reduced the incidence of ischemic stroke nor increased the proportion of the independent population in the surgery and non-surgery groups. As the subgroup analysis included few studies, the results should be treated with caution.

### 4.2. Limitations

This study had some limitations that should be considered before recommending these findings to clinical practice. First, the subgroup analysis was limited, possibly due to the relatively different clinical backgrounds of the populations and the limited number of studies. For example, of the three studies that included hemorrhagic stroke, one included patients who underwent revascularization, one included patients who did not undergo revascularization for at least 6 months during the initial clinical follow-up, and one did not report anything. In addition, some studies show that, unlike the Asian population, MMD progresses slowly but relatively well in non-Asian populations such as the Caucasian population (28, 29). Therefore, subgroup analysis should be performed according to the population to explore the benefits and risks of APT. Limited by the inclusion and exclusion criteria, the studies included in this systematic review were all performed in Asia, so it was unclear whether the benefits and risks of APT differ between Asian and non-Asian MMD patients. Secondly, although the Newcastle Ottawa scale was used to objectively evaluate the quality of studies and the results indicated that the included studies were of moderate to high quality, selection bias, information bias, and confounding bias may still exist because of the inherent limitations of cohort studies (30). Third, the outcome evidence from long-term randomized controlled trials was ideal; however, studies in this area have not been conducted. Meta-analyses from high-quality cohort studies may be a potentially powerful approach to comprehensively evaluate the benefits and risks of APT for MMD. Fourth, the observation period of the included study varies greatly, and the operation time point was not reported, which all may affect the outcome. Although we used HR to solve the secondary heterogeneity of follow-up time difference in statistical analysis, it is far from enough.

### 4.3. Future perspectives

To address this unmet clinical need for treating MMD, high-quality prospective cohort studies or randomized controlled trials are needed in the future. To date, there are no ongoing prospective studies evaluating the benefits and risks of APT in MMD on the Clinical Trials Registry platform. Considering whether or not to undergo surgery is an important covariate when evaluating the efficacy of APT in patients with MMD, the surgical and non-surgical populations should be separately included in future studies to evaluate the relationship between APT and long-term outcomes.

## 5. Conclusion

In this systematic review, APT reduced the risk of hemorrhagic stroke but did not reduce the risk of ischemic stroke or increase the proportion of independent patients. There was insufficient evidence about the benefit of APT on survival and postoperative bypass patency after surgical revascularization. However, the results should be interpreted with caution because of the limited number of studies.

## Data availability statement

The original contributions presented in the study are included in the article/Supplementary material, further inquiries can be directed to the corresponding authors.

## Author contributions

YG and TLiu: study conception and design. TLiu: systematic literature search and statistical analysis. MQ and XX: study screening, data extraction, and risk of bias assessment. TLiu and XL: drafting the manuscript. YG, XL, and LF: supervision. All authors: critical revision of the manuscript for important intellectual content. All authors contributed to the article and approved the submitted version.
