# Supplementary material for: Benefits and risks of antiplatelet therapy for moyamoya disease: a systematic review and meta-analysis
Source: Front Neurol. 2023 Jun 20;14:1132339. doi: 10.3389/fneur.2023.1132339 (PMC10318533; doi:10.3389/fneur.2023.1132339)
Supplement: Supplementary file 1 [file Data_Sheet_1.doc]

Supplementary Material

**Benefits and Risks of Antiplatelet Therapy for Moyamoya Disease: A Systematic Review and Meta-analysis**

**Tingting Liu^1, 2, 3^, Mingzhen Qin^1, 2, 3^,** **Xuejiao Xiong^1, 2, 3^****, Tingting Li^1, 2, 3^, Luda Feng^4^, Xinxing Lai****^1, 2 *^, Ying Gao^1, 2,5*^**

^1^ Institute for Brain Disorders, Beijing University of Chinese Medicine, Beijing 100700, China

^2^ Department of Neurology, Dongzhimen Hospital, Beijing University of Chinese Medicine, Beijing 100700, China

^3^ Beijing University of Chinese Medicine, Beijing 100029, China

^4^ Dongfang Hospital, Beijing University of Chinese Medicine, Beijing 100078, China.

^5^ Chinese Medicine Key Research Room of Brain Disorders Syndrome and Treatment of the National Administration of Traditional Chinese Medicine, Beijing 100700, China

*** Correspondence:**

**Ying Gao**

Institute for Brain Disorders, Beijing University of Chinese Medicine

Department of Neurology, Dongzhimen Hospital, Beijing University of Chinese Medicine

Beijing 100700, China

E-mail: gaoying973@163.com

**Xinxing Lai**

Institute for Brain Disorders, Beijing University of Chinese Medicine

Department of Neurology, Dongzhimen Hospital, Beijing University of Chinese Medicine

Beijing 100700, China

E-mail: [new-star@163.cm](mailto:new-star@163.cm)

**Table S1** Search Strategy Example: PubMed search

**Fig. S1.** Forest plot for subgroup analysis of ischemic stroke based on surgery or not

**Fig. S2.** Forest plot for subgroup analysis of ischemic stroke based on literature quality

**Fig. S3.** Forest plot for sensitivity analysis of ischemic stroke

**Fig. S4.** Forest plot for sensitivity analysis of hemorrhagic stroke

**Fig. S5.** Forest plot for subgroup analysis of the proportion of independent patients based on surgery or not

**Fig. S6.** Forest plot for subgroup analysis of the proportion of independent patients based on literature quality

**Fig. S7.** Forest plot for sensitivity analysis of the proportion of independent patients

**Table S1** Search Strategy Example: PubMed search

| **No** | **Search items** |  |
| --- | --- | --- |
| 1 | Moyamoya Disease |  |
| 2 | Progressive Intracranial Occlusive Arteropathy (Moyamoya) |  |
| 3 | Moyamoya Syndrome |  |
| 4 | Moya-Moya Disease |  |
| 5 | Disease, Moya-Moya |  |
| 6 | Moya Moya Disease |  |
| 7 | Disease, Primary Moyamoya |  |
| 8 | Disease, Classic Moyamoya |  |
| **9** | **1 OR 2-8** |  |
| 10 | Platelet Aggregation Inhibitors |  |
| 11 | antiplatelet agent* |  |
| 12 | anti-platelet agent* |  |
| 13 | antiplatelet therap* |  |
| 14 | anti-platelet therap* |  |
| 15 | antiplatelet drug* |  |
| 16 | anti-platelet drug* |  |
| 17 | antiplatelet medication* |  |
| 18 | anti-platelet medication* |  |
| 19 | platelet aggregation inhibit* |  |
| 20 | antithrombotic agent* |  |
| 21 | anti-thrombotic agent* |  |
| 22 | antithrombotic therap* |  |
| 22 | anti-thrombotic therap* |  |
| 23 | antithrombotic drug* | |
| 24 | anti-thrombotic drug* | |
| 25 | antithrombotic medication* | |
| 26 | anti-thrombotic medication* | |
| **27** | **10 OR 11-26** | |
| 28 | Aspirin | |
| 29 | Picotamide | |
| 30 | acetylsalicylic acid | |
| 31 | aggrenox | |
| 32 | Purinergic P2Y Receptor Antagonists | |
| 33 | Clopidogrel | |
| 34 | Prasugrel Hydrochloride | |
| 35 | Ticagrelor | |
| 36 | Cangrelor | |
| 37 | Ticlopidine | |
| 38 | Elinogrel | |
| 39 | P2Y12 receptor antagonis* | |
| 40 | adenosine reuptake inhibit* | |
| 41 | adenosine re-uptake inhibit* | |
| 42 | adenosine diphosphate receptor inhibit* | |
| 43 | Phosphodiesterase Inhibitors | |
| 44 | Cilostazol | |
| 45 | Dipyridamole | |
| 46 | phosphodiesterase inhibit* | |
| 47 | Adenosine Diphosphate Antagonis* | |
| 48 | Adenosine Diphosphate Inhibit* | |
| 49 | Abciximab | |
| 50 | Tirofiban | |
| 51 | Eptifibatide | |
| 52 | Beraprost | |
| 53 | Ditazole | |
| 54 | Platelet Glycoprotein GPIIb-IIIa Complex Inhibit* | |
| 55 | Platelet Glycoprotein GPIIb-IIIa Complex Antagonis* | |
| 56 | glycoprotein IIB/IIIA inhibitors | |
| 57 | **28 OR 29-56** | |
| 58 | **9 AND (27 OR 57)** | |

**

**

**Fig. S1.** Forest plot for subgroup analysis of ischemic stroke based on surgery or not





**Fig. S2.** Forest plot for subgroup analysis of ischemic stroke based on literature quality





**Fig. S3.** Forest plot for sensitivity analysis of ischemic stroke





**Fig. S4.** Forest plot for sensitivity analysis of hemorrhagic stroke





**Fig. S5.** Forest plot for subgroup analysis of the proportion of independent patients based on surgery or not





**Fig. S6.** Forest plot for subgroup analysis of the proportion of independent patients based on literature quality





**Fig. S7.** Forest plot for sensitivity analysis of the proportion of independent patients
